# No free entry: stomatal state as decision maker in defining stress response strategies

**DOI:** 10.1093/jxb/erae447

**Published:** 2025-01-08

**Authors:** Łukasz P Tarkowski, Santiago Signorelli

**Affiliations:** INRAE, Université de Strasbourg, UMR SVQV, Colmar, France; Food and Plant Biology Group, School of Agriculture, Universidad de la República, Montevideo, 12900, Uruguay; School of Molecular Sciences, University of Western Australia, Crawley, WA 6009,Australia

**Keywords:** Defence priming, herbivory, multi-stress, stomata, VOCs

## Abstract

This article comments on:

**Maleki FA, Seidl-Adams I, Felton GW, Kersch-Becker MF, Tumlinson JH**. 2024. Stomata: gatekeepers of uptake and defense signaling by green leaf volatiles in maize. Journal of Experimental Botany **75**, 6872–6887. https://doi.org/10.1093/jxb/erae401.

This article comments on:

**Maleki FA, Seidl-Adams I, Felton GW, Kersch-Becker MF, Tumlinson JH**. 2024. Stomata: gatekeepers of uptake and defense signaling by green leaf volatiles in maize. Journal of Experimental Botany **75**, 6872–6887. https://doi.org/10.1093/jxb/erae401.

**Stomatal openings are valves constituting the main aerial interface between plants and the surrounding environment. Therefore, the regulation of stomatal conductance is essential for plants to adapt their physiology to any sort of environmental cues. The study by Maleki *et al*. (2024) sheds light on the role of stomata in the uptake of volatile danger signals, and hence in their perception and signal transduction. These findings consolidate the importance of stomatal regulation in reaction to environmental stresses and demonstrate how stomatal closure can interfere with the intra- and inter-plant signals that modulate biotic stress responses.**

## Stomatal conductance regulation enables plants to counteract most types of stress

The evolutionary trajectory of the plant kingdom is marked by taking the direction towards a sessile lifestyle, which demanded a series of ingenious and fascinating adaptations to withstand environmental conditions. Despite lacking freedom of movement, the development of a phenomenal biochemical and physiological elasticity led land plants to the evolutionary success that we can observe nowadays, allowing them to colonize almost every biotope, accounting for 80% of biomass on earth. Stomata, microscopic pores in the leaf and stem epidermis, are arguably one of the pillars of such success. They form a dense network of microscopic adjustable valves that constitute the primary interface between plant aerial parts and the surrounding environment, allowing for biophysical parameters such as gas exchange, leaf temperature, and water potential to be finely tuned as a reaction to environmental stimuli (Lawson and Morison, 2004). Stomatal function is therefore vital primarily to respond to abiotic stresses, drought, and heat. However, the importance of stomatal regulation encompasses responses to biotic stresses as well, since both pathogens and defence-associated signals can enter through these gates. On the one hand, a vast body of evidence supports the concept of stomatal defence as a mechanism that regulates stomatal conductance, following pathogen and herbivory perception, to limit their progression (Melotto *et al.*, 2017). Conversely, pathogens have evolved molecular tools to evade this defence and access the nutrient-rich apoplastic environment and beyond (Wu and Liu, 2022). On the other hand, stomatal opening is a mandatory condition for receiving and perceiving different VOCs (volatile organic compounds) produced either by nearby plants or different leaves within the same plant under attack to activate defence priming (Wang and Erb, 2022). This mechanism creates a stress memory in plants exposed to a first stress episode, allowing them to respond in a faster and stronger manner to a subsequent stress event. Defence priming is typically characterized by a maintenance phase between the two stress episodes, where the plant ‘prepares’ its cells for future attacks by adopting a series of molecular adjustments. This maintenance phase is typically characterized by low energetic costs, making defence priming an advantageous strategy, notably in environments with high disease pressure (Box 1) (Martinez-Medina *et al.*, 2016).

## Defence priming induced by herbivores is subordinated to stomatal conductance regulation

Herbivore-induced plant volatiles (HIPVs) are probably the best-characterized class of defensive VOCs, with maize being the model plant to study their perception and role. HIPV perception triggers a stress memory that results in subsequent accumulation of the phytohormone jasmonic acid (JA), a main player in immune responses to herbivory, and several defence-associated terpenes (Aguirre *et al.*, 2023; Waterman *et al.*, 2024). This HIPV-dependent defence priming is inhibited by stomatal closure triggered by the decline of photosynthetic activity during night-time and abscisic acid (ABA) signalling, which can be elicited by abiotic cues such as drought (Aguirre *et al.*, 2023). This illustrates how plants integrate different environmental stimuli (circadian, biotic, or abiotic) through stomata to optimize the use of their resources (growth–defence trade-off). The novel study of Maleki *et al*. (2024) goes further in dissecting the interaction among these mechanisms. By performing experiments with different doses of an exogenous HIPV (*Z*-3-hexen-1-ol) and exposure times, notably with regular pulses to simulate herbivory feeding, the authors show that stomata opening is required for HIPV uptake and that this is neither time nor dose dependent. The finding that dark-induced stomatal closure limits HIPV uptake, no matter their concentration or duration of exposure, suggests that plants prioritize certain environmental stimuli over others to fine-tune their growth–defence trade-off (Maleki *et al.*, 2024). This study further shows that HIPV-induced biosynthesis of sesquiterpenes, molecules to attract natural enemies of herbivores, is inhibited following stomatal closure. This inhibition is not reversed by the application of herbivorous larvae regurgitant (a defence elicitor), suggesting that the establishment of the primed state requires HIPV uptake through stomata regardless of the perception of herbivory (Maleki *et al.*, 2024).

## Stomatal regulation at the basis of resource optimization

The study by Maleki *et al*. (2024), together with the previous report by Aguirre *et al*. (2023), reveals a pivotal role for stomatal regulation in coordinating the hierarchy of environmental stimuli that can be divided into three categories: (i) defence-related signals (e.g. HIPVs); (ii) light-dependent signals; and (iii) abiotic stress signals (especially those involving ABA response). These two studies are consistent in indicating light perception as a more dominant stimulus in stomatal regulation when compared with defence-related signals. Thus, light-dependent stomatal regulation appears to determine resource management by inhibiting perception of volatile danger signals at night-time, therefore avoiding the perturbation of the plant physiological and biochemical ‘activity schedule’ imposed by the circadian clock, responsible for circadian fluctuations in almost all metabolite families present in plants (Venkat and Muneer, 2022). The synthesis of complex secondary metabolites induced by HIPVs, such as sesquiterpenes, has high metabolic costs which are harder to sustain during the night than during the day due to the lack of the photosynthetic energy supply (Gershenzon, 1994). Several reports have shown that a number of defensive compounds and signals have circadian fluctuations. Notably, JA accumulation was shown to peak during the day in Arabidopsis, and this was linked to a selective advantage for plants against herbivory attacks (Goodspeed *et al.*, 2013). Stomatal regulation may thus provide an additional level of control over defence expenditure to optimize metabolic resource management. However, herbivory can induce stomatal closure and interfere with HIPV perception in attacked plants, hence subverting host stomatal regulation and resulting in a decreased capacity for defence induction (Lin *et al.*, 2021). Similarly, ABA-induced stomatal closure in response to abiotic stress was shown to be more dominant than the defence-related HIPV stimulus (Aguirre *et al.*, 2023; Maleki *et al.*, 2024).

**Fig. 1.**
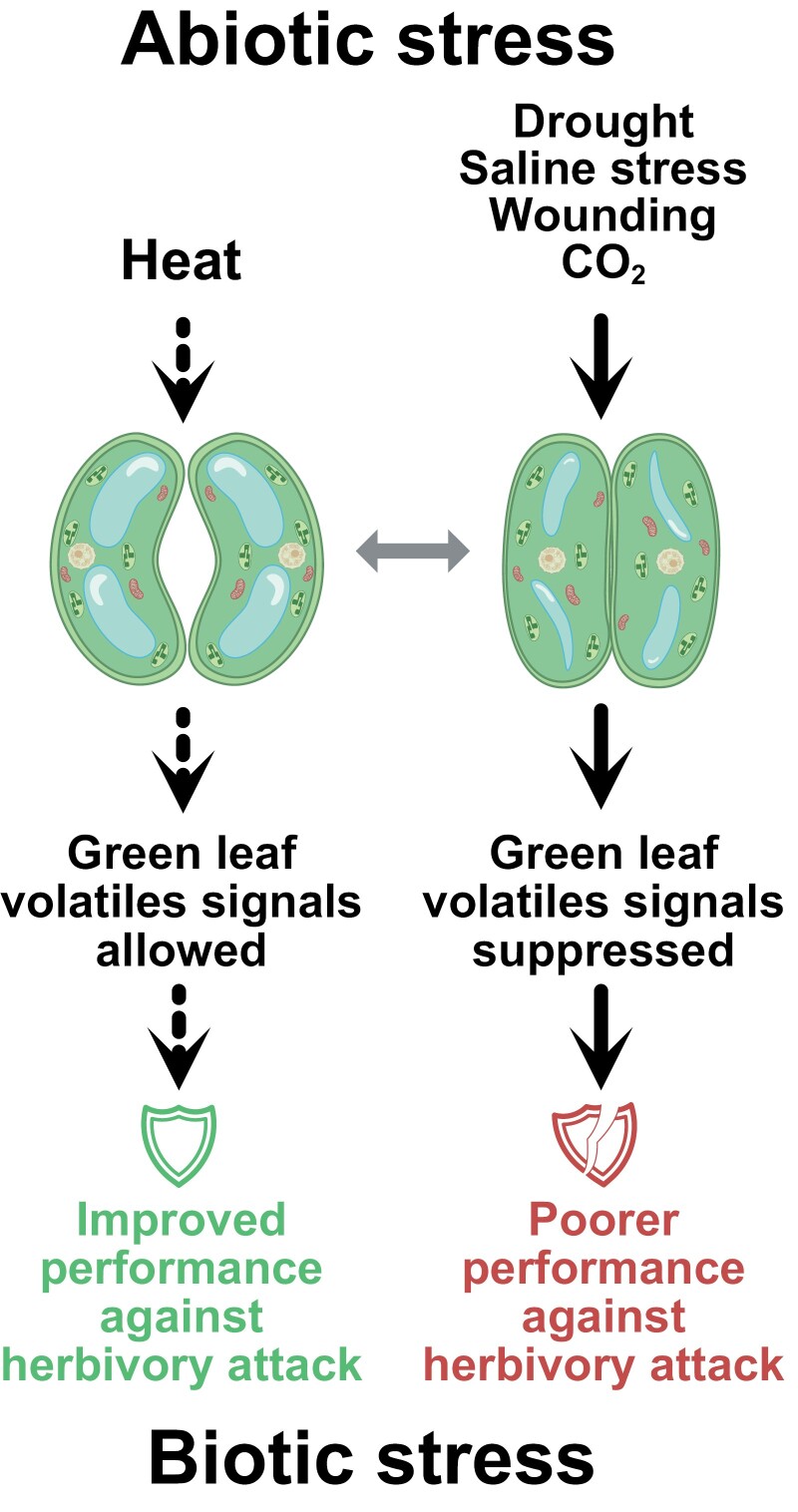
Effect of abiotic stress-induced stomatal aperture modulation on biotic (herbivory) stress responses mediated via volatile organic compounds. Abiotic stress can induce stomatal aperture changes, resulting in receptivity or insensitivity towards volatile signals. Volatile-triggered defence induction ultimately improves resistance to herbivory attack. Dotted lines indicate hypothetical effects.

Taken together, defence priming mediated by HIPVs appears to be heavily dependent on environmental conditions, so much so that in a situation of abiotic and biotic stress combination, the presence of the abiotic stress can completely abolish the responses meditated by HIPVs and thus compromise the plant performance against herbivores (Fig. 1). Such subordination may be linked to the high metabolic cost of the compounds induced by priming. Interestingly, several volatiles comparable with HIPVs can prime against abiotic stress as well (Sako *et al.*, 2020). Volatile-induced secondary metabolites that can alleviate both biotic and abiotic stress might counterbalance their energetic costs through their multifunctionality in a highly stressful environment.

## Perspectives: integrating resistance to multiple stresses through stomatal regulation

**Fig. 2.**
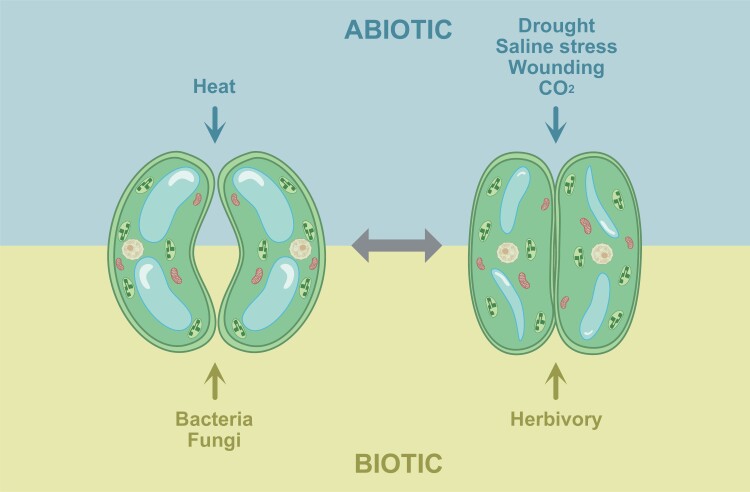
Schematic summary of the effects of abiotic and biotic stresses on stomatal conductance. Water deficit caused by drought or salinity as well as high CO_2_ trigger stomatal closure, while increased temperature induces stomatal opening. Several bacterial and fungal pathogens can force stomatal opening that enables microbes to penetrate inside the leaf, while herbivores can induce stomatal closure which reduces emission of volatiles that attract their predators.

**Fig. 3.**
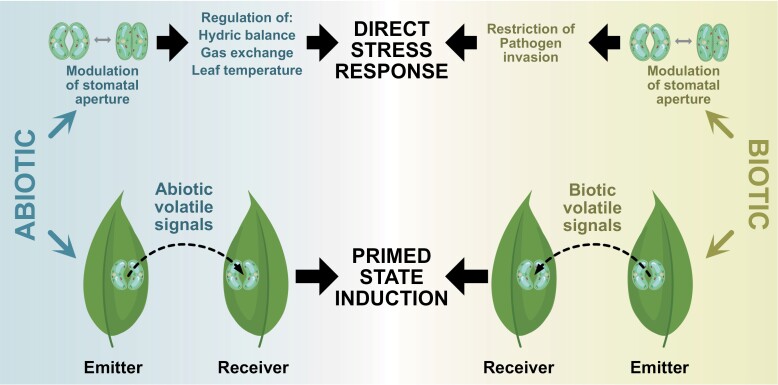
Role of stomata in direct and indirect responses to abiotic and biotic stresses. The stomatal opening state orchestrates stress responses by acting at two levels: directly (upper part) by regulating leaf physical parameters and accessibility to inner tissues, and indirectly (lower part) by determining receptivity towards volatile signals that are able to induce priming.

Generating knowledge about plant responses to multiple stresses is an indispensable step to develop crop protection strategies effective under field conditions (Zandalinas *et al.*, 2024). The regulation of stomatal conductance is an essential parameter involved in multi-stress tolerance. However, interpretating its global impact on plant fitness is not straightforward due to antagonistic effects that stomatal closure/openness have on several biotic–abiotic stress combinations (Fig. 2). For instance, elevated foliar temperature due to heat stress can induce stomatal opening, while potentially promoting leaf invasion by pathogens. Other combinations, such as drought and pathogens, can result in enhanced biotic resistance due to decreased pathogen penetration rate (Gao *et al.*, 2018). Thus, although most of the abiotic–biotic stress combinations provoke cumulative damage to plants, some of them can result in increased resistance under certain circumstances (Pandey *et al.*, 2024). In this sense, a critical point to investigate is how simultaneous signals from different stresses are integrated to modulate stomatal state. Experiments featuring a range of different inoculum/infestation pressures combined with different abiotic stress intensities would help in understanding this integration. Complementarily, understanding whether volatile signals filtered by stomata can prime against different categories of stresses at once would provide a comprehensive picture of stomatal functions in direct and indirect responses to multiple stresses (Fig. 3). Such research is expected to improve our understanding of the mechanisms governing resource optimization under multiple environmental cues, ultimately generating data with an immediate and reliable applicative value. Considering the increasing speed of climate change and the difficulties in transposing a large portion of results generated in controlled conditions to the field, the urgency to generate such knowledge must be emphasized.

Box 1. **Defence priming against biotic stress**

Experimental evidence sustaining the induction of a stress memory in plants came from early works of Uwe Conrath and colleagues in the 1990s, with experiments based on exogenous application of phytohormones or their analogues (Conrath *et al.*, 1995). This paved the way to intense research in the following two decades, resulting in an extensive comprehension of this phenomenon (Martinez-Medina *et al.*, 2016). Different classes of signals associated with biotic stress were demonstrated to be capable of eliciting defence priming (Mauch-Mani *et al.*, 2017). This includes phytohormones at low doses (high doses would trigger direct defence activation), endogenous molecules such as amino acids, or molecular patterns directly associated with biotic aggression, typically divided into MAMPs, HAMPs, and DAMPs (microbe-, herbivore-, and danger-associated molecular patterns, respectively). While the first two are associated with molecular components derived from specific biotic stressors, the last one is associated with molecules produced from, or released by, damage to host tissues inflicted by stressors. These signals are typically perceived and transduced via receptor kinases at the cell surface to trigger biological processes that help plant cells to ‘prepare for the battle’ in case of future attack. Although such processes are yet to be fully understood, some of them have been relatively well characterized and exemplify the high degree of adaptability of plants at the molecular level when confronted with stressful conditions. Noteworthy cases are the modulation of chromatin accessibility to regions carrying defence genes, and the accumulation of proteins involved in defence signalling in their inactive form, such as in the case of the mitogen-activated protein kinases MAPK3 and MAPK6 (Beckers *et al.*, 2009; Sheikh *et al.*, 2023).
